# Treatment of mites folliculitis with an ornidazole-based sequential therapy: A randomized trial

**DOI:** 10.1097/MD.0000000000004173

**Published:** 2016-07-08

**Authors:** Yang Luo, Yu-Jiao Sun, Li Zhang, Xiu-Li Luan

**Affiliations:** Department of Dermatology, Lanzhou General Hospital of Lanzhou Military Area Command, Lanzhou, China.

**Keywords:** betamethasone, mites folliculitis, ornidazole, recombinant bovine basic fibroblast growth factor, sequential therapy

## Abstract

**Objective:**

Treatment of *Demodex* infestations is often inadequate and associated with low effective rate. We sought to evaluate the efficacy of an ornidazole-based sequential therapy for mites folliculitis treatment.

**Methods:**

Two-hundred patients with mites folliculitis were sequentially treated with either an ornidazole- or metronidazole-based regimen. Sebum cutaneum was extruded from the sebaceous glands of each patient's nose and the presence of *Demodex* mites were examined by light microscopy. The clinical manifestations of relapse of mites folliculitis were recorded and the subjects were followed up at 2, 4, 8, and 12 weeks post-treatment.

**Results:**

Patients treated with the ornidazole-based regimen showed an overall effective rate of 94.0%. Additionally, at the 2, 4, 8, and 12-week follow-up, these patients had significantly lower rates of *Demodex* mite relapse and new lesion occurrence compared with patients treated with the metronidazole-based regimen (*P* < 0.05).

**Conclusion:**

Sequential therapy using ornidazole, betamethasone, and recombinant bovine basic fibroblast growth factor (rbFGF) gel is highly effective for treating mites folliculitis.

## Abstract 2

Supplemental Digital Content is available in the text

## 1 Introduction

Mites folliculitis is an inflammation of skin hair follicles and surrounding tissue resulting from infestation with *Demodex*, a common human ectoparasite that is found in about 10% of skin biopsies and 12% of hair follicles.^[1–3]^*Demodex* infestations occur in 23% to 100% of healthy adults,^[1]^ while nearly 100% of the elderly population reportedly carries *Demodex* in their skin follicles; additionally, the elderly tend to harbor a larger variety of mites.^[4,5]^ Although *Demodex* infestations are usually asymptomatic, resultant changes in the immune system can cause numerous types of dermatological conditions. While *Demodex* infestation mainly manifests as cutaneous inflammation, it can also generate suppurative or granulomatous lesions that result in acne,^[6]^ rosacea^[7–9]^ or perioral dermatitis.^[10]^ Additionally, *Demodex folliculorum* has been implicated in various types of papular and pustular eruptions on the head and neck, including demodicosis and rosacea types.^[11]^

The most common treatment of *Demodex* infestations is metronidazole.^[12–14]^ Topical metronidazole administered in combination with azelaic acid and oral doxycycline is effective for treating moderate to severe rosacea, which is another cutaneous disease associated with *Demodex* infestation.^[13]^ However, the effective rate associated with this type of combined treatment is low because of poor compliance. O*rnidazole* is a 5-nitroimidazole compound similar to metronidazole, and is used as an antiamebic agent. O*rnidazole* has a longer biological half-life than metronidazole, and produces fewer side-effects in patients. In a previous study,^[15]^ ornidazole was shown to have better efficacy (both parasitological and clinical) than metronidazole in treating patients with dientamoebiasis.^[15]^ However, while metronidazole is a well-known and commonly used anti-protozoal agent, the acaricidal effect and clinical efficacy of ornidazole in treating mites folliculitis have not been previously evaluated.

Additionally, topical use of agents for *Demodex* infestations is not always feasible, as it may cause irritation in patients with sensitive skin. In a preliminary study, we observed that ornidazole administration was associated with aggravated inflammation at lesion sites starting at 4 days post-treatment. Thus, following the ornidazole treatment, we applied compound betamethasone injection (CBI), based upon the potent glucocorticoid compound with anti-inflammatory properties, coupled with recombinant bovine basic fibroblast growth factor (rbFGF) gel; the combination therapy shortened healing time, and improved the quality of wound healing.

## 2 Methods

### 2.1 Patients

**Figure 1:**
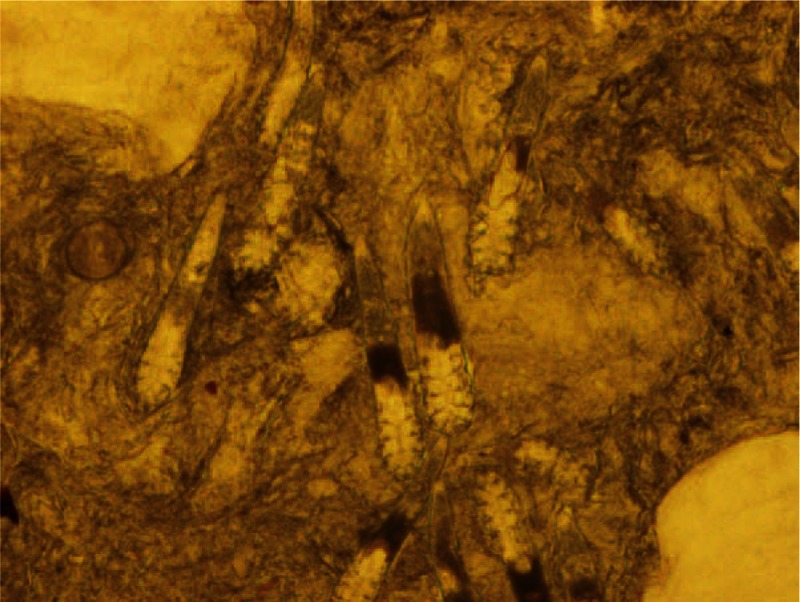
Checked for the live mites by microscopy. Original magnification, ×40.

Patients diagnosed with mites folliculitis by clinical and/or histopathologic examination in the dermatological clinics of the Lanzhou General Hospital of Lanzhou Military Region were recruited from May 2014 to Nov 2014. Estimation of the study's sample size was based on the minimum number of cases used in previous clinical trials of metronidazole therapy, aiming for an expected error value of 10%; when the level of significance was set as *P* = 0.05 and the confidence interval was set 95%, the sample size was determined to be 96. A brief outline of the study design is shown in Fig. 1.

**Figure 2:**
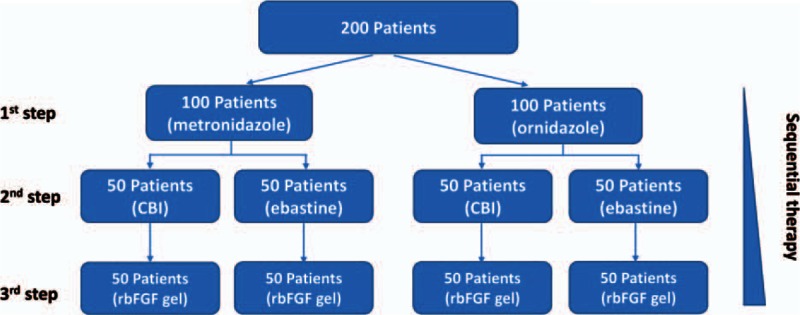
Schematic diagram showing the grouping and treatment of patients with mites folliculitis.

A total of 200 patients (151 women and 49 men; 20–45 years of age; mean disease duration of 2 months) were enrolled in this study. Baseline demographic and clinical characteristics of patients in each group are shown in Table 1. All patients had no history of drug allergy, tumors, or a diagnosis and treatment of mites folliculitis. The women patients had normal menstruation. All enrolled patients had their facial skin checked for the presence of live mites by microscopy (Fig. 2). The study protocol was approved by the Ethics Committee of Lanzhou General Hospital of Lanzhou Military Region, and a signed Informed Consent form was obtained from each patient prior to enrolment.

**Table 1:**
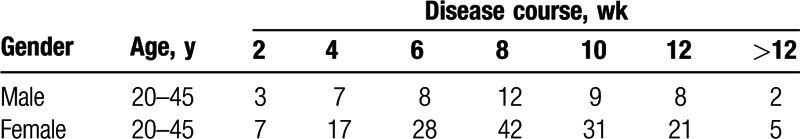
Baseline characteristics and disease course for the patients enrolled in this study.

### 2.2 Grouping and randomization method

A single-blind, parallel, unicenter, randomized clinical trial was carried out. Patients who fulfilled the inclusion criteria were randomly allocated to two groups according to random number tables. The patients were blinded to their treatment drugs and measures were taken to ensure that such a single-blinded protocol would not lead to assessment bias. Randomization was then performed by assigning the random numbers from the random number tables to the two treatments. This strategy aimed to prevent selection bias and helped to ensure against accidental bias. To achieve allocation concealment, the staff members who were involved in assigning the random numbers to patients were excluded from the process of selecting numbers.

### 2.3 Treatments, follow-up and outcomes

The enrolled patients were randomly assigned to two study groups (n = 100 each). One group received ornidazole (0.5 g/time, t.d., per os) while the other group received metronidazole (0.2 g/time, q.d.s., per os) for 14 days. After 4 days of treatment, 50 patients in each group were randomly assigned to receive a single dose of CBI (1 mL, i.m.). Only a single dose was injected, because the effects of CBI last up to 3 weeks. The remaining 50 patients in each group were treated with ebastine (10 mg, o.d., per os) for 3 weeks. To alleviate skin lesions induced by the antibiotic treatment, topical rbFGF gel was applied to the lesions (1 g, t.d.) for 14 consecutive days beginning on day 7 post-ornidazole or -metronidazole treatment. Following rbFGF gel treatment, patients in the group treated with CBI received no further therapy, while patients in the group treated with ebastine continued antihistamine for 1 additional week. After completing respective treatments, the subjects in both groups were followed-up during clinical visits at 2, 4, 8, and 12 weeks post-treatment.

The primary outcomes were effective rates achieved after 2 weeks of treatment with the 2 different regimens and relapse rates. We also observed the presence and severity of skin lesions and inflammation during the treatment period. The following surrogate endpoints were set and patients were either denied enrollment or advised to withdraw from the study (i.e., stop the study-related treatment) in each of the following situations: experience of a situation that disagrees with the original inclusion criteria; significant risk of safety; no significant improvement of health; non-compliance with the treatment strategy.

### 2.4 Detection of demodex mites

*Demodex* infestation was diagnosed by the microscopic examination of sebum cutaneum extruded from sebaceous glands by squeezing both sides and the tip of the nose. Following extrusion, individual specimens were scraped off the nose with the back end of a dip pen point. Each sebum specimen was then diluted with a drop of glycerin and placed onto glass slide and examined under a light microscope for the presence of living mites. This process was repeated each week during the course of treatment to evaluate the efficacy of therapy.

### 2.5 Statistical analysis

All data were analyzed using IBM SPSS Statistics for Windows, Version 19.0. (Armonk, NY: IBM Corp.). Efficacy was defined as a remission of existing skin lesions and the absence of any new lesions. Chi-square test was used to compare the efficacies ornidazole and metronidazole after 2 weeks of treatment. Differences in the efficacies of ornidazole- and metronidazole-based regimens for preventing recurrence of *Demodex* mite infestations and formation of new lesions after an initial treatment were compared using survival analysis. *P* values <0.05 were considered statistically significant.

## 3 Results

### 3.1 Skin lesions and inflammation after ornidazole or metronidazole treatment

**Figure 3:**
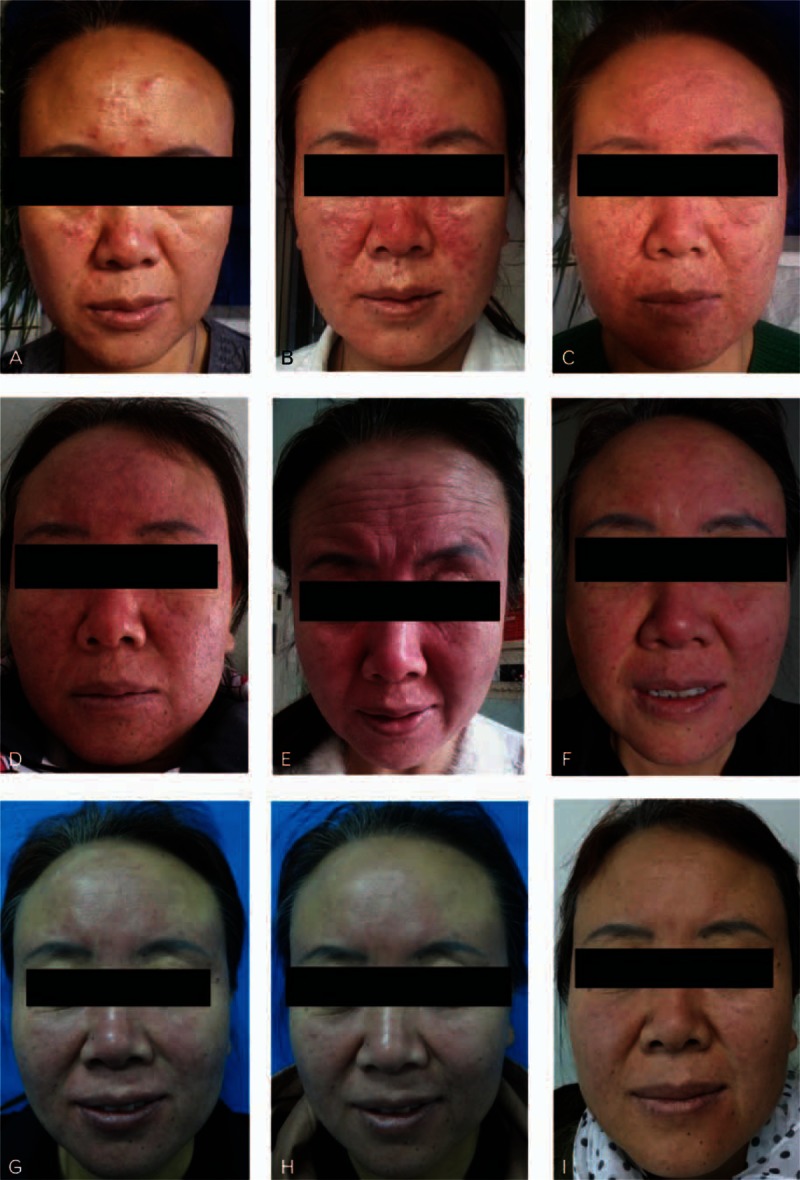
Effect of different treatments on the morphologic presentation of mites folliculitis. A: 6 weeks before treatment; B: 4 weeks before treatment; C: 2 weeks before treatment; D: 1 day before treatment; E: 4 days post-ornidazole treatment; F: 3 days post-CBI treatment; G: 1 week after rbFGF gel treatment; H: 2 weeks after rbFGF gel treatment; I: 6 weeks after ornidazole treatment. CBI = compound betamethasone injection, rbFGF = recombinant bovine basic fibroblast growth factor.

**Figure 4:**
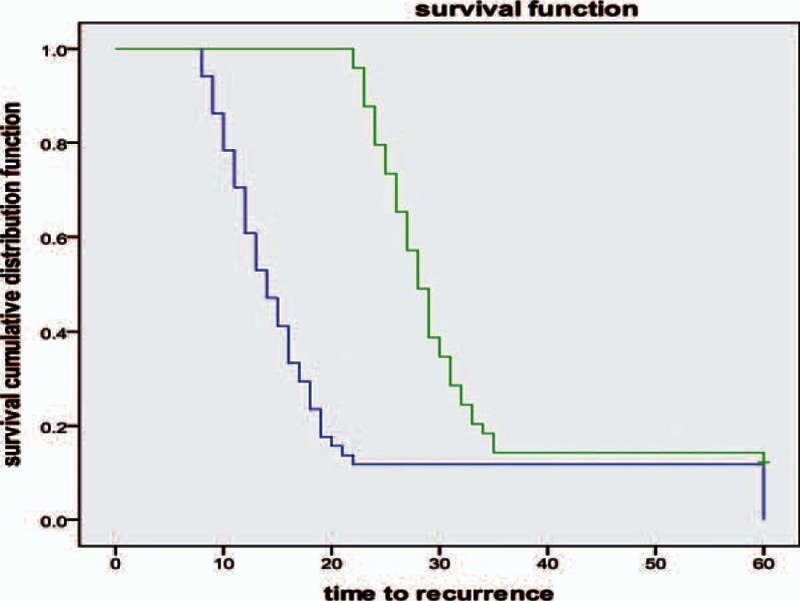
Survival analysis of *Demodex* mites after sequential therapy with ornidazole in combination with CBI or ornidazole in combination with ebastine. Blue line = ornidazole + CBI, Green line = ornidazole + ebastine. *P* < 0.0001. CBI = compound betamethasone injection.

**Figure 5:**
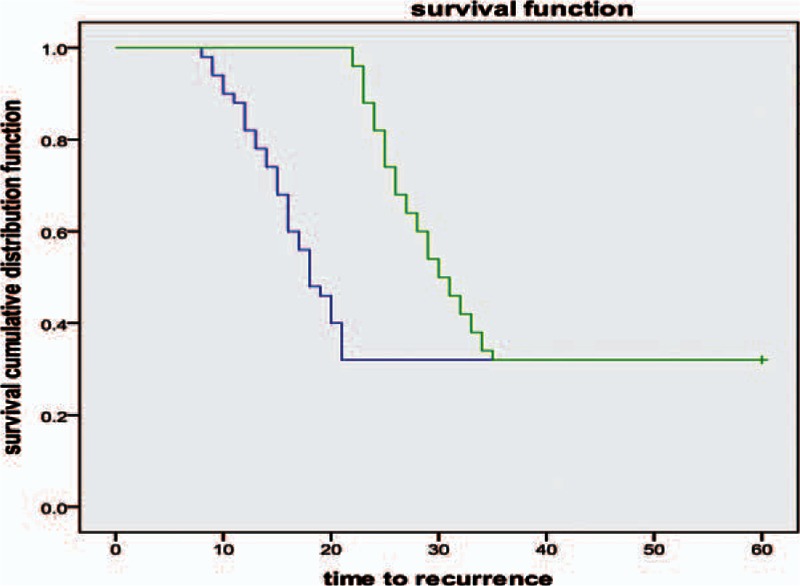
Survival analysis of *Demodex* mites after sequential therapy with metronidazole in combination with CBI or metronidazole in combination with ebastine. Blue line = metronidazole + CBI, Green line = metronidazole + ebastine. *P* < 0.0001. CBI = compound betamethasone injection.

Both ornidazole- or metronidazole-treated patients showed remarkable clinical improvement. Representative pictures of a woman patient at various time points prior to treatment are shown in Fig. 3A–D. Mites folliculitis initially manifested as 0.3-cm papula located on the forehead and nose. The skin lesions were initially centered on the nose, and then gradually spread across the entire face. The facial inflammation became aggravated following treatment with either ornidazole or metronidazole (Fig. 3E), suggesting the increased occurrence of foreign body reactions following death of the *Demodex* mites. However, inflammation was significantly alleviated by treatment with CBI (Fig. 3H), which had better efficacy (Figs. 4 and 5) than ebastine (data not shown). The skin lesions were later healed by topical use of rbFGF gel. As shown in Fig. 4, the two combined treatment regimens (ornidazole + CBI + rbFGF gel or ornidazole + ebastine + rbFGF gel) demonstrated similar efficacies in mite killing and remission of folliculitis; however, the required lengths of the treatment periods for two regiments were significantly different. Combined treatment with ornidazole plus CBI and rbFGF gel showed a more rapid effect in inhibition of mite reproduction, and also for relieving symptoms of itching and skin inflammation when compared with treatment with ebastine plus rbFGF gel (Fig. 3G–H). There was almost no skin inflammation at 6 weeks post-treatment (Fig. 3I).

### 3.2 Comparison of therapeutic outcomes achieved using ornidazole or metronidazole-based treatments

We next compared the effective rates achieved after 2 weeks of treatment with the two different regimens. Two weeks of treatment with ornidazole plus CBI and rbFGF gel produced an overall effective rate of 94.0%, which was significantly higher than that achieved using metronidazole plus CBI and rbFGF gel (*x*^2^ = 10.631, *P* = 0.002) (Table 2). Furthermore, similar therapeutic efficacies were observed in men and women patients (*P* > 0.05). However, there were no statistical differences observed between the therapeutic efficacies of the two treatments for the men patients, although the effective rates for both were similar to the efficacies for the overall population and for the women patients; although, this finding may be because of the small sample size of men patients.

**Table 2:**
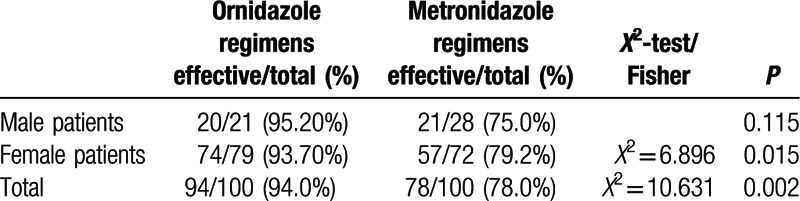
Comparison of effective rates after 2-weeks of treatment with an ornidazole or metronidazole-based regimen.

Relapse is always a problem in treating demodicosis. The two groups in our study were distinguishable by their respective rates of relapse, as diagnosed during follow up visits. Patients treated with the metronidazole-based regimen had a higher relapse rate (26, 34, 36, and 37 cases at 2, 4, 8, and 12 weeks post-treatment, respectively) compared with patients treated with the ornidazole-based regimen (6, 12, 13, and 15 cases at 2, 4, 8, and 12 weeks post-treatment, respectively; all *P* values ≤0.001) (Table 3). Additionally, patients treated with the metronidazole-based regimen had a higher rate of new lesion occurrence following treatment: (22, 30, 32, and 33 cases at 2, 4, 8, and 12 weeks post-treatment, respectively, in the metronidazole group vs. 6, 12, 13, and 13 patients at 2, 4, 8 and 12 weeks post-treatment, respectively, in the ornidazole group; *P* < 0.01) (Table 4).

**Table 3:**
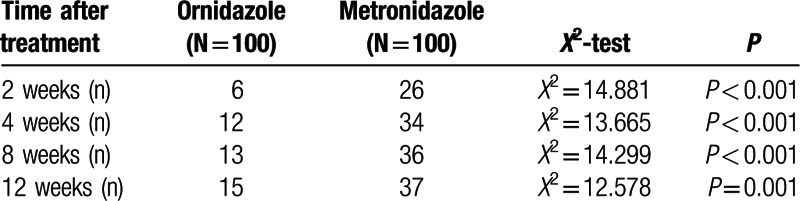
Comparison of *Demodex* mite relapse post-treatment with an ornidazole- or metronidazole-based regimen.

**Table 4:**
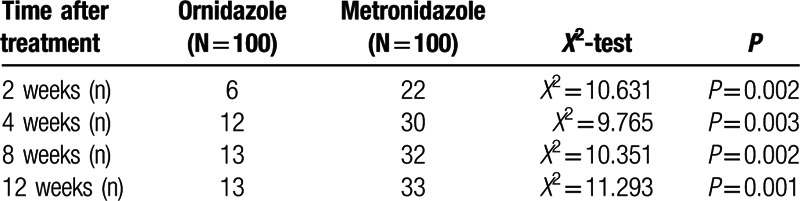
Comparison of new lesion occurrence post-treatment with an ornidazole- or metronidazole-based regimen.

## 4 Discussion

In the present study, we evaluated the efficacy of an ornidazole-based combined sequential regimen in treating mite folliculitis, and compared the results with those obtained when using a standard combined metronidazole-based regimen. Our results showed that the ornidazole-based regimen (sequential treatments with ornidazole, CBI, and topical rbFGF gel) was highly effective in reducing *Demodex* counts, curing the infestation, suppressing mite relapse, and preventing the occurrence of new lesions.

Follicular mites can be found in patients with a variety of unrelated skin disorders^[2]^ and the prevalence increased in elderly individuals.^[2,4]^ Several pharmacologic agents are currently available for treating mites folliculitis, including metronidazole, selenium sulfide,^[12]^ ortho-hydroxybenzoic acid,^[16]^ and gammexane.^[16,17]^ Although metronidazole has shown better efficacy than the other three agents, its systemic use is associated with numerous side-effects that limit its use in the clinic. Combined treatment of *D folliculorum* with camphor oil and metronidazole has been reported to alleviate itching in humans.^[18]^ Furthermore, systemic administration of ivermectin is reported to reduce the numbers of *D folliculorum* found in the eyelashes of patients with refractory blepharitis, especially in cases that were previously unsuccessfully treated because of poor compliance.^[19]^ Ivermectin rapidly cleared papulopustular dermatosis of the scalp and granulomatous rosacea,^[20]^ and another study showed that pilocarpine gel reduced the numbers of mites and alleviated itching,^[21]^ probably because of the direct toxicity to mites, as its muscarinic activity is known to impede respiration and motility.^[21]^ However, the successful use of these agents requires long treatment times, during which side-effects or disease aggravation can occur; resulting in poor compliance and treatment failure.

Ornidazole is a 5-nitroimidazole derivative that has antimicrobial activity conveyed by its nitro group, and is widely used in treatment of vaginal trypanosomiasis, amoebosis, and other diseases caused by protozoans. In an anaerobic environment, the nitro group of ornidazole can be chemically reduced to an amino group or to a free radical that reacts with the components of microorganisms, resulting in microbial death. Thus far, there have been no reports concerning the use of ornidazole for treating mites folliculitis. A previous study showed that a single dose of ornidazole demonstrated better efficacy (both parasitologically and clinically) than metronidazole in treating patients with dientamoebiasis.^[15]^ Furthermore, o*rnidazole* has a longer biological half-life and fewer side-effects than metronidazole. The longer half-life of ornidazole allows for a twice-daily dosing regimen, *which is associated with better patient compliance*. In contrast, metronidazole must be orally administered four times per day. Finally, ornidazole produces fewer side-effects than metronidazole, which also helps to improve patient compliance with prescribed dosing regimens.

In a preliminary study, we found that ornidazole significantly increased the degree of inflammation at folliculitis lesion sites. Some patients had to discontinue therapy because of severe inflammatory response as manifested by inflammation that involved the entire face. This exacerbated inflammation was at least partially caused by the foreign body reaction induced by the rapid death of *Demodex* mites exposed to ornidazole, and the resulting accumulation of parasite waste products and polypides. Therefore, in the present study, we first treated patients with ornidazole for 4 days, and then administered CBI as the second step. Betamethasone is a potent glucocorticoid with anti-inflammatory properties, and is widely used to treat itching associated with various pathological conditions.^[14,22]^ CBI can be administered by intramuscular injection when treating patients with *Demodex*-associated cutaneous diseases and who have sensitive skin, and thus we selected this dosage form for use in our current study. As expected, CBI significantly alleviated the facial lesion inflammation and itching that occurred following treatment with ornidazole (Fig. 3F); however, treatment with ebastine (a non-sedating antihistamine used to treat allergic conditions), produced little effect.

**Figure 6:**
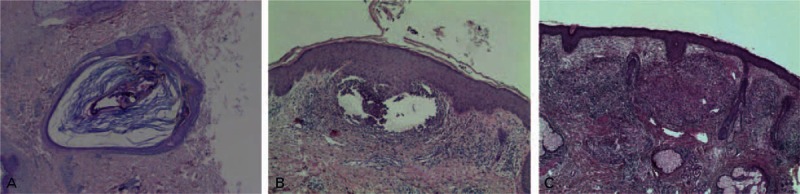
Hematoxylin-eosin staining of the skin biopsy. A: *Demodex* mite in the hair follicle. B: Mites folliculitis formation under the inflammatory lesion skin; C: A granuloma formation under the inflammatory lesion skin. Original magnification, ×40.

Finally, mites folliculitis usually occurs on the face. However, as the life cycle of mites is short, it is difficult to detect inflammatory lesions at the sites with living mites (Fig. 6A). In contrast, tissues with mite residues around often exhibit inflammation (Fig. 6B) where no clearly identifiable mite can be observed. A granuloma formation under the inflammatory lesion skin is shown in Fig. 6C. It is thus conceivable that mites folliculitis would be induced by the exposure of residues or the polypide contents to the tissues and the host immune response. In further study, we will check whether there are any folliculitis cases with more clearly identifiable mite. For treating the inflammation, following CBI injection, we applied bovine rbFGF gel for repair of the facial lesions. rbFGF is a potent mitogen, a chemoattractant for endothelial cells, fibroblasts, and keratinocytes, and is commonly applied externally to promote the healing of wounds and chronic surface ulcers. In previous studies conducted by other investigators, rbFGF decreased wound healing time and improved wound healing quality in patients with burns, donor sites, or chronic dermal ulcers.^[23–25]^ In our study, application of rbFGF topical gel significantly improved the repair of skin lesions during both the course of treatment and follow-up as compared with patients without rbFGF use (not included in this report).

Our current study has several limitations. First, this is a single blind study. Second, when treating facial lesions associated with *Demodex* mites, we did not continuously monitor the pathological status of the dermis (Fig. 6). Third, the mechanism by which ornidazole kills *Demodex* mites remains unclear and requires further investigation.

In conclusion, a combination regimen of ornidazole, CBI, and rbFGF gel administered in a sequential manner effectively killed *Demodex* mites, and also alleviated facial inflammation, reduced itching symptoms, and induced the rapid repair of skin lesions. Thus, this novel sequential therapy represents a promising treatment for mites folliculitis.

## Supplementary Material

Supplemental Digital Content
