# Supplementary material for: Treatment of mites folliculitis with an ornidazole-based sequential therapy: A randomized trial
Source: Medicine (Baltimore). 2016 Jul 8;95(27):e4173. doi: 10.1097/MD.0000000000004173 (PMC5058870; doi:10.1097/MD.0000000000004173)

Clinical trial protocol and statistical analysis plan

Objective

To assess the efficacy of an ornidazole-based sequential therapy for the treatment of mites folliculitis.

Inclusion criteria

Normal liver functions; normal menstruation for female patients. All patients had facial sebum cutaneum squeezed from lesion and checked under microscope of living mites.

Exclusion criteria

History of drug allergic to metronidazole, ornidazole, Compound Betamethasone Injection, ebastine, or recombinant bovine basic fibroblast growth factor; prior diagnosis and treatment of mites folliculitis; <20 years old or >45 years old; pregnant or lactation women; patients with severe primary diseases in cardiovascular, liver, kidney or hematopoietic systems; patients with mental disorder; patients with tumor; menstrual abnormalities/disorders or history of menopause for female patients.

Ethics

The study protocol was approved by the Ethics Committee of Lanzhou General Hospital of Lanzhou Military Region, and a signed Informed Consent form was obtained from each patient prior to enrollment. (Clinicaltrial registration number: ChiCTR-IPR-15006451).

Patients

A single-blind, parallel, unicenter, randomized clinical trial was carried out. Patients who fulfill the inclusion criteria were checked by clinical and/or histopathologic examination in the dermatological clinics of the Lanzhou General Hospital of Lanzhou Military Region were recruited from May 2014 to Nov 2014. Estimation of sample size is based on the minimum number of cases used in previous clinical trials using metronidazole therapy that the expected error value would be 10%. The level of significance was set as P=0.5 and confidence interval set 95% in the study. Thus, the sample size for the study would be 96. That means we would need to complete approximately 96 patients each group for the study. A total of 200 patients (151 females and 49 males; 20-45 years of age) with a mean disease duration of 2 months were enrolled in this study. These patients were randomly allocated to two groups according to random number tables with consent.

Grouping and randomization method

The patients were blind to their treatment drugs and measures were taken to ensure that such a single-blind would not lead to assessment bias. Randomization was then performed by assigning the random numbers from random number tables to the two treatments. This strategy prevents the selection bias and insures against the accidental bias. To achieve allocation concealment, the staff members that involved in assigning random numbers to patients were excluded from selecting numbers.

Treatment

The enrolled patients were randomly assigned to two study groups (n=100 each). One group (79 female, 21 male) received ornidazole (0.5 g/time, t.d., *per os*) while the other group (72 female, 28 male) received metronidazole (0.2 g/time, q.d.s., *per os*) for 14 days. After four days of treatment, 50 patients in each group were randomly assigned to receive a single dose of CBI (1 mL, *i.m.*). The remaining 50 patients in each group were treated with ebastine (10 mg, o.d., *per os*) for 3 weeks. Topical rbFGF gel was applied to the lesions (1 g, t.d.) for 14 consecutive days beginning on day 7 post-ornidazole or -metronidazole treatment. Following rbFGF gel treatment, patients in the group treated with CBI received no further therapy, while patients in the group treated with ebastine continued antihistamine for one additional week. After completing respective treatments, the subjects in both groups were followed-up during clinical visits at 2, 4, 8, and 12 weeks post-treatment.

Content

The study protocol was approved by the Ethics Committee of Lanzhou General Hospital of Lanzhou Military Region, and a signed Informed Consent form was obtained from each patient prior to enrollment.

End points

The following surrogate endpoints for patients were set and patients will not be enrolled or advised to quit in each of the following situation: 1) not fulfill the inclusion criteria; 2) significant risk of safety issue; 3) no significant improvement of health; 3) don't follow the guidance; 4) no strict compliance of treatment strategy.

Indices of efficacy

1. Clinical observation

Remission of facial skin lesions; mite numbers.

2. Histopathological examination

Biopsy of skin tissues for *histopathological examination with consent*

Statistical and effective rate analysis plan

1. Patients

The enrolled patients were randomly assigned to two study groups (n=100 each). One group (79 female, 21 male) received ornidazole (0.5 g/time, t.d., *per os*) while the other group (72 female, 28 male) received metronidazole (0.2 g/time, q.d.s., *per os*) for 14 days. After four days of treatment, 50 patients in each group were randomly assigned to receive a single dose of CBI (1 mL, *i.m.*). The remaining 50 patients in each group were treated with ebastine (10 mg, o.d., *per os*) for 3 weeks. Topical rbFGF gel was applied to the lesions (1 g, t.d.) for 14 consecutive days beginning on day 7 post-ornidazole or -metronidazole treatment. Following rbFGF gel treatment, patients in the group treated with CBI received no further therapy, while patients in the group treated with ebastine continued antihistamine for one additional week. After completing respective treatments, the subjects in both groups were followed-up during clinical visits at 2, 4, 8, and 12 weeks post-treatment.

2. Effective rate analysis

All the effective effect analysis will be based on an intention-to-treat population or per-protocol population method.

3. ***Adverse reactions and Relapse analysis***

In case of severe adverse reactions with either treatment, the patient will be advised to quit the research and subjected to corresponding remedies accordingly.

4. Statistical method

All data were analyzed using IBM SPSS Statistics for Windows, Version 19.0. (Armonk, NY: IBM Corp). Efficacy was defined as a remission of existing skin lesions and the absence of any new lesions. Chi-square test was used to compare the efficacies ornidazole and metronidazole after two weeks of treatment. Differences in the efficacies of ornidazole- and metronidazole-based regimens for preventing recurrence of *Demodex* mite infestations and formation of new lesions after an initial treatment were compared using survival analysis. *P*-values < 0.05 were considered statistically significant.

5. Software and data presentation

The IBM SPSS Statistics for Windows, Version 19.0. (Armonk, NY: IBM Corp) will be used for statistical analysis, and data will be presented as mean ± standard deviation (SD) or percentage where appropriate.

Research plan and timelines:

1. May 5, 2014 – Nov 1, 2014: patient recruitment
2. May 5, 2014 – Feb 26, 2015: Treatment, pathological examination; follow-up, pathological examination
3. Mar, 2015 – Organizing the data collected and performing preliminary analysis of data.
4. Sep 2015 – Completing the clinical data collection.

Figure 1. Flow diagram showing the patient enrollment, allocation, follow-up and analysis in this study

Estimation of sample size is based on the minimum number of cases used in previous clinical trials using metronidazole therapy that the expected error value would be 10%. The level of significance was set as P=0.5 and confidence interval set 95% in the study. Thus, we would need to complete approximately 96 patients each group for the study. A brief outline of the study design is shown in **Fig. 1**.

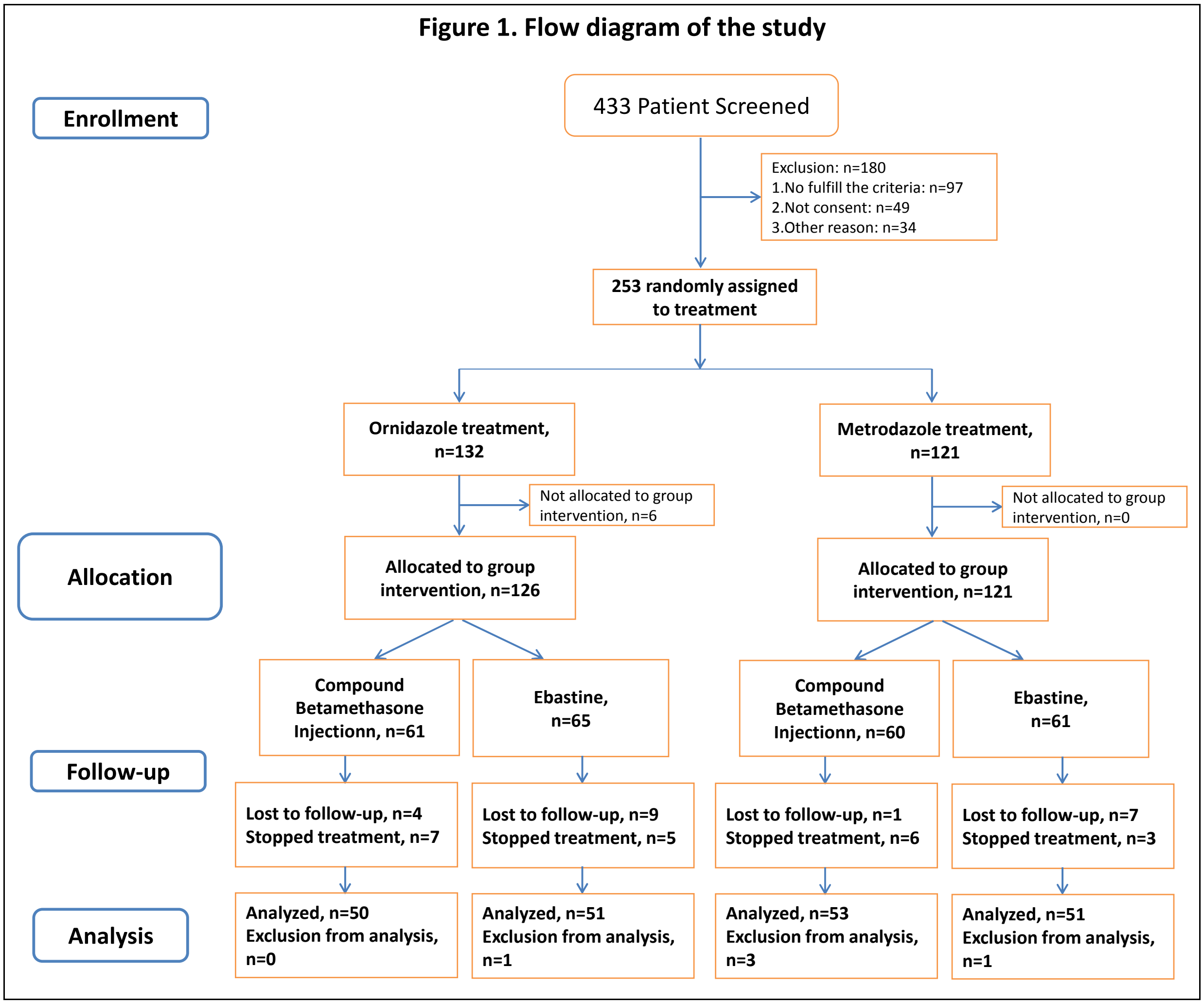

Supplement: Supplemental Digital Content [file medi-95-e4173-s001.pdf]
